# The Effect of Simple Cost Effective Interventions in Improving Enhanced Recovery in Neck of Femur Fracture Care

**DOI:** 10.7759/cureus.11217

**Published:** 2020-10-28

**Authors:** Elizabeth Wilson, Arta Vala, Jamie O'Callaghan, Philip McCann, Khalid Al-Hourani

**Affiliations:** 1 Trauma and Orthopaedics, Bristol Royal Infirmary, Bristol, GBR

**Keywords:** enhanced recovery programme, neck of femur fractures, orthopaedics surgery, best practice tariff, orthogeriatrics, orthopaedics trauma

## Abstract

Aim

Due to the frequency and high mortality and morbidity associated with neck of femur fractures, pathways of care have been established in the United Kingdom. These include the Enhanced Recovery Program (ERP), which aims to maximise the quality of care whilst reducing their length of stay, and the Best Practice Tariff (BPT) which if adhered to warrants £1335 per neck of femur fracture. We conducted a prospective audit to assess adherence to these pathways in a trauma unit.

Methods

An audit was carried out between November 2015 and May 2016. The information was obtained from neck of femur fracture proformas, anaesthetic charts and drug charts by two investigators.

Results

Nine out of the 10 ERP components were adhered to in all 31 patients. This highlighted a deficiency in requesting day one post-operative osteoporosis bloods, which was only carried out in 61.3% of patients. As an intervention, a reminder sticker was placed on the operation note as an intervention. Re-audit following the introduction of the stickers showed a marked improvement of 90%. During the initial admission 38.7% of patients adhered to the BPT. The main area for improvement was fracture prevention assessment, specifically Fracture Risk Assessment Tool (FRAX) scores and Nottingham Hip Fracture Scores. To improve this these sections were highlighted in the proformas to promote their importance. Additionally, a smartphone application was made available to doctors to aid with ease of calculation. Following these interventions, 93% of patients had this data entered, with an improvement in overall tariff attainment to 63.3%.

Conclusions

The introduction of simple measures is beneficial both for patient safety and economically for hospitals.

## Introduction

In the United Kingdom, neck of femur fractures represent the most frequently seen traumatic injury and the commonest reason for admission to an orthopaedic ward in the elderly population [1]. In 2018, hospitals in the UK treated almost 70,000 patients with neck of femur fractures [2] and this is likely to increase in line with the aging population. Predictions made by joint guidance issued by the British Geriatrics Society (BGS) and the British Orthopaedic Association (BOA) estimate that the caseload will double by 2020 [1]. Patients sustaining a neck of femur fracture are associated with high mortality and morbidity rates due to the frail, multi-morbid nature of many of these patients. Additionally, neck of femur fractures represent a significant financial burden for the National Health Service, costing an estimated £1.1 billion per year [3]. Most of these costs are incurred during the first year post-injury [3]. In response to these issues, measures were introduced by healthcare bodies such as the BOA, the BGS and the National Institute of Health and Care Excellence (NICE), in order to improve patient outcomes from the outset [4]. This is complemented by The National Hip Fracture Database, established in 2007, as a joint venture between the BOA and the BGS, and tracks key indicators in neck of femur fracture patients, including length of stay, 30-day mortality and attainment of the Best Practice Tariff (BPT).

The BPT outlines set criteria which emphasise the importance of prompt surgery and the early involvement of orthogeriatricians [5]. Full attainment of the Tariff criteria earns £1,335 for the Trust per patient. In those for whom the criteria are not met, there is a £435 deduction per patient, resulting in a base earning of £900 per patient. Three new criteria were introduced to the BPT in 2017, which dictate that in addition to the pre-existing criteria, patients should receive a nutritional assessment, a delirium assessment using the 4AT tool, and a physiotherapy assessment either the day of surgery or the day following [5].

The other main pathway currently utilised in the treatment of patients with neck of femur fractures is the Enhanced Recovery Pathway (ERP). The ERP originated in Denmark and aims to maximise the quality of care patients receive whilst simultaneously reducing their length of stay [6]. Enhanced recovery pathways are now used across multiple different surgical specialties in both emergency and elective procedures. They have been shown to reduce morbidity, result in early postoperative mobilization and reduced readmission rates [7,8]. This is achieved through the optimisation of patient care during their hospital stay using a set of criteria. These criteria emphasize the prescription of appropriate medication during admission, including analgesia on admission, pre-operative and post-operative nutritional supplements, laxatives, and postoperative prophylactic antibiotics. It also focuses on bone health, requiring day one post-operative bone profile blood tests, and the prescription of secondary prevention medication for osteoporosis if aged over 75 years. We conducted a prospective audit aiming to assess adherence to these standards in patients admitted with neck of femur fractures to the treating trauma unit.

## Materials and methods

A prospective closed loop audit was carried out to assess the adherence to the ERP and BPT in a dedicated trauma unit from November 2015 - May 2016. The standards used in this analysis were the Best Practice Tariff and the NHS Enhanced Recovery Partnership Programme Guidelines. The criteria for these in our unit for hip fracture patients can be found in Table 1 and Table 2, respectively. Ethical approval was sought from the department in accordance with local policies. Two investigators (EW, AV) collated the data and input it into a database on secure National Health Service computers to protect patient confidentiality. The data was obtained from the neck of femur admission proformas of patients admitted to the orthopaedic unit. This was cross-referenced with data from anaesthetic charts, drug charts, and the Trust’s electronic results system. 

**Table 1:** Hip Fracture Best Practice Tariff Criteria 2016

Time to surgery within 36 hours from arrival into ED to the start of anaesthesia
Admitted under the joint care of a consultant geriatrician and consultant orthopaedic surgeon
Admitted using an assessment protocol agreed by geriatricians, orthopaedic surgeons and anaesthetics
Assessment by a geriatrician in the peri-operative period: within 72 hours of admission
Documentation of pre-operative and post-operative abbreviated mental score
Postoperative geriatrician guided multiprofessional rehabilitation team
Fracture prevention assessments (bone and falls assessments)

**Table 2:** Enhanced Recovery Programme Criteria

Appropriate analgesia on admission
Prescription of pre-operation carbohydrate drink
Prescription of post-operation fortisips if concerned regarding nutrition
Prescription of laxatives
Prescription of prophylactic antibiotics post surgery
Prescription of osteoporosis medication if aged over 75
Osteoporosis bloods requested day one post operation

Patients over the age of 60 years old with a diagnosis of a fractured neck of femur were included in the study. Those with a periprosthetic fracture or pathological fracture were excluded from the study. 

**Figure 1:**
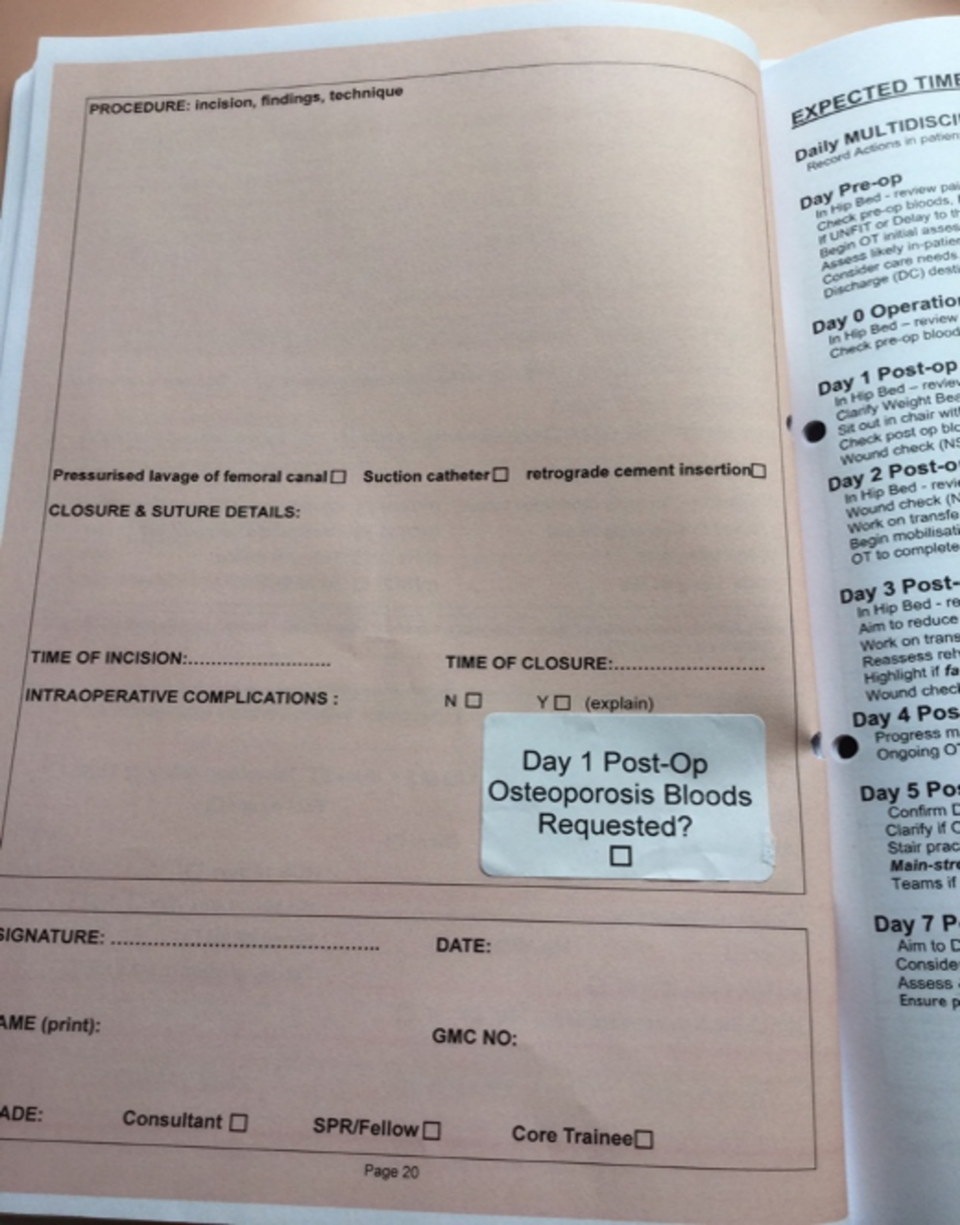
Photograph of reminder sticker placed in operation note

The initial audit period lasted for two months. After completion, the results were reviewed and validated by two other authors (JOC, KAH) and three simple interventions were implemented by the Trauma & Orthopaedic department. Firstly, admitting physicians in the department were educated as to the importance of completing the hip fracture scores. This was via formal written reminders as well as presenting the results of the cycle one in the local morbidity and mortality meeting. This included emphasis on the Nottingham Hip Fracture Score (NHFS) and its use as a tool for accurately predicting 30-day mortality, and the Fracture Risk Assessment Tool (FRAX), which can be used to predict risk of future fractures. The second intervention consisted of a modification to the existing hospital smartphone application to incorporate the scoring systems. Admitting teams were then recommended to download this to their phones. The third intervention was a reminder sticker that was placed on the operation note page within the hip fracture proforma to request the osteoporosis profile bloods day one postoperatively, and for this to be done on the electronic system in the immediate post-operative (for the next day) period along with the operation note (Figure 1).

Following the implementation of these interventions, the second cycle of the audit was carried out, allowing for a two-week acclimatisation period. The standards used were the same as those used for the initial cycle and the inclusion and exclusion criteria remained the same. 

## Results

The first cohort had a total of 31 patients with a mean age of 82 years (SD=11.49) and a male female ratio of 2:3. Of these, no patients (n=0) had a FRAX or NHFS recorded during their admission. The proportion of patients who had osteoporosis bloods requested at day one post-operatively was 61.3% (n=19). The delay in results of blood tests directly correlated with a delay in commencement of secondary bone protective medication. All 31 patients had 100% attainment of the remaining ERP parameters. The full BPT criteria were met in only 38.7% (n=12) of patients, with bone and falls health assessment highlighted as key areas of deficiency. The financial losses due to the number of patients in which the BPT was not achieved during this period was £8,265.

**Figure 2:**
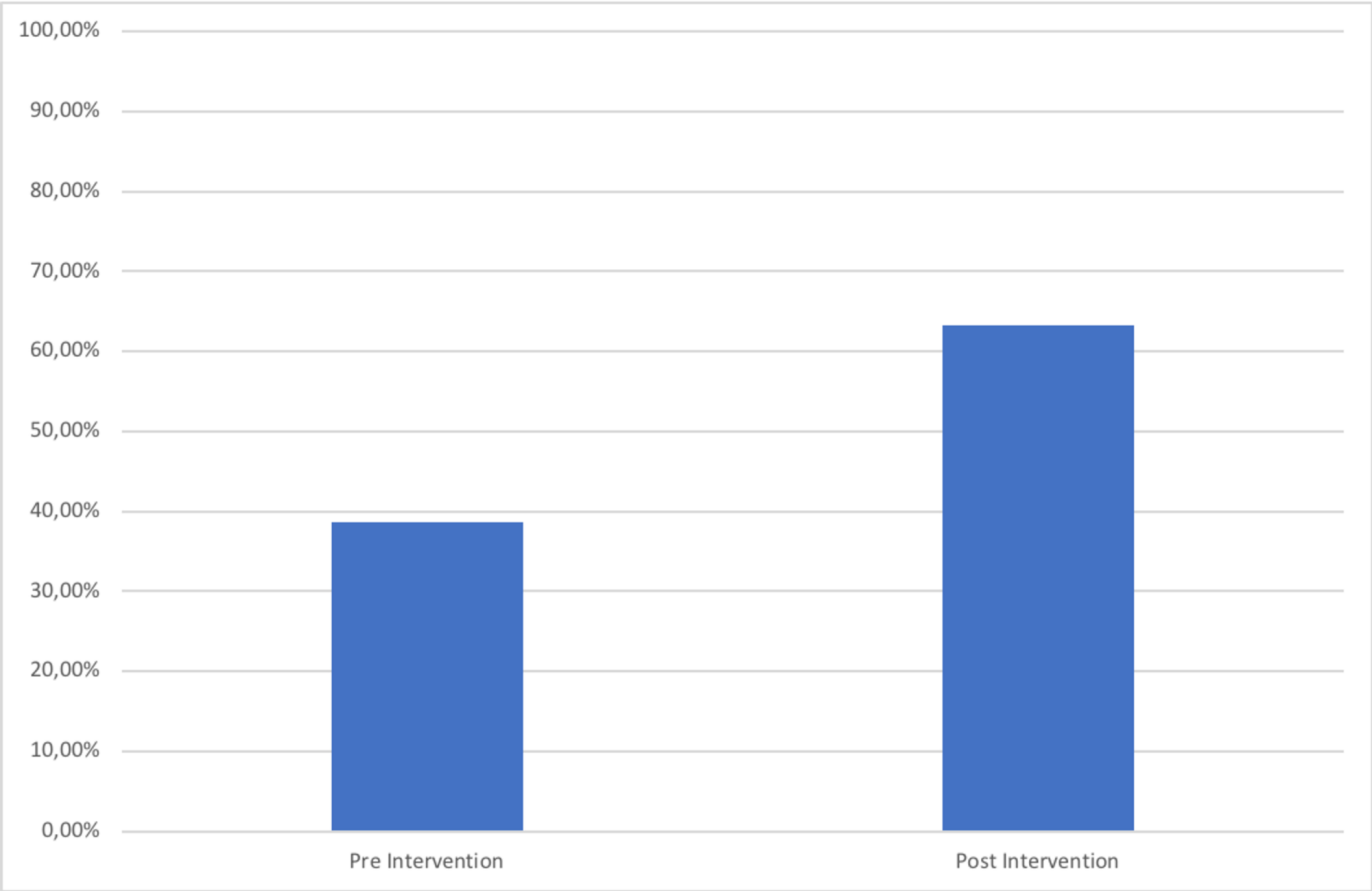
Percentage of patients achieving the Best Practice Tariff Criteria in each audit cycle

**Figure 3:**
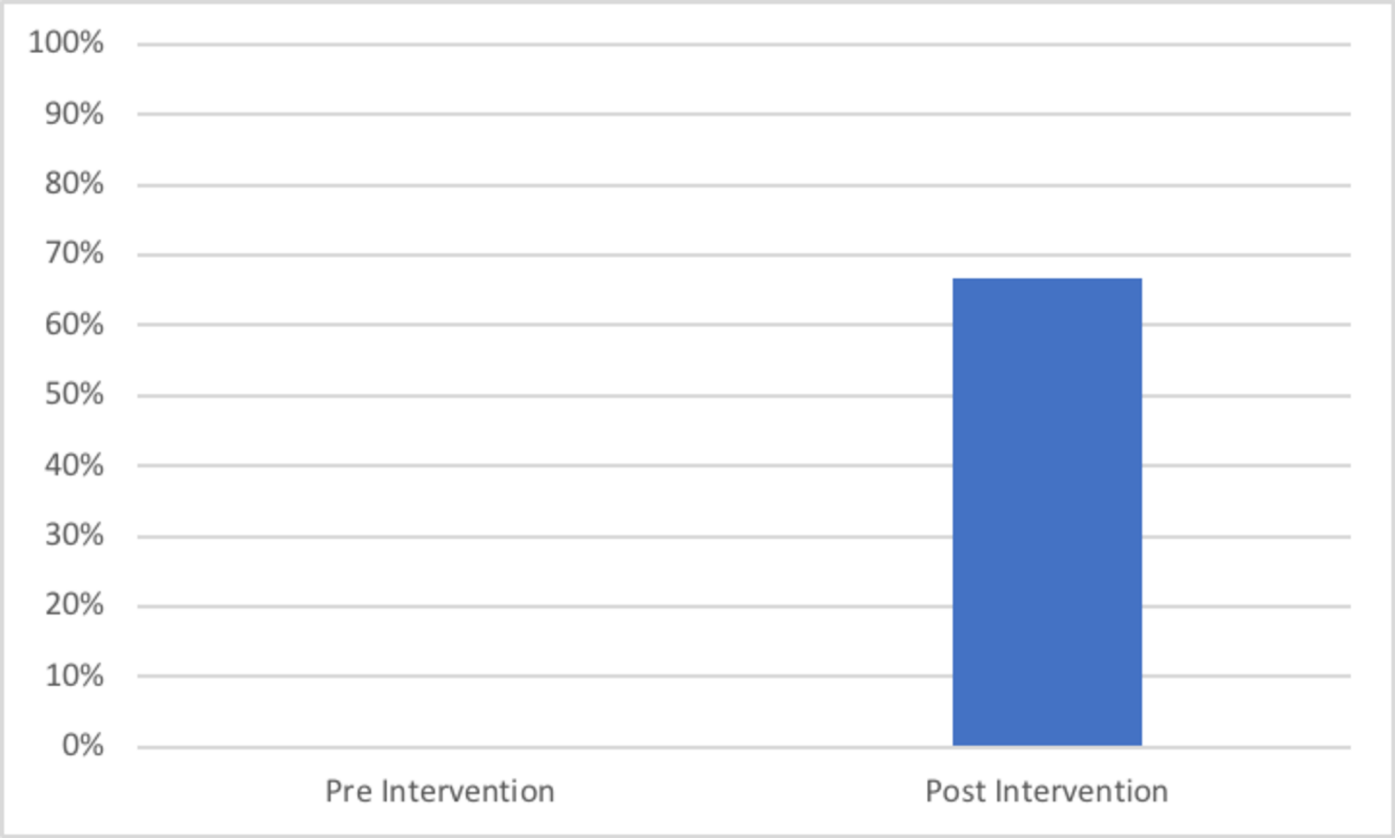
Percentage of patients in each cycle who had a Nottingham Hip Fracture Score (NHFS) calculated

**Figure 4:**
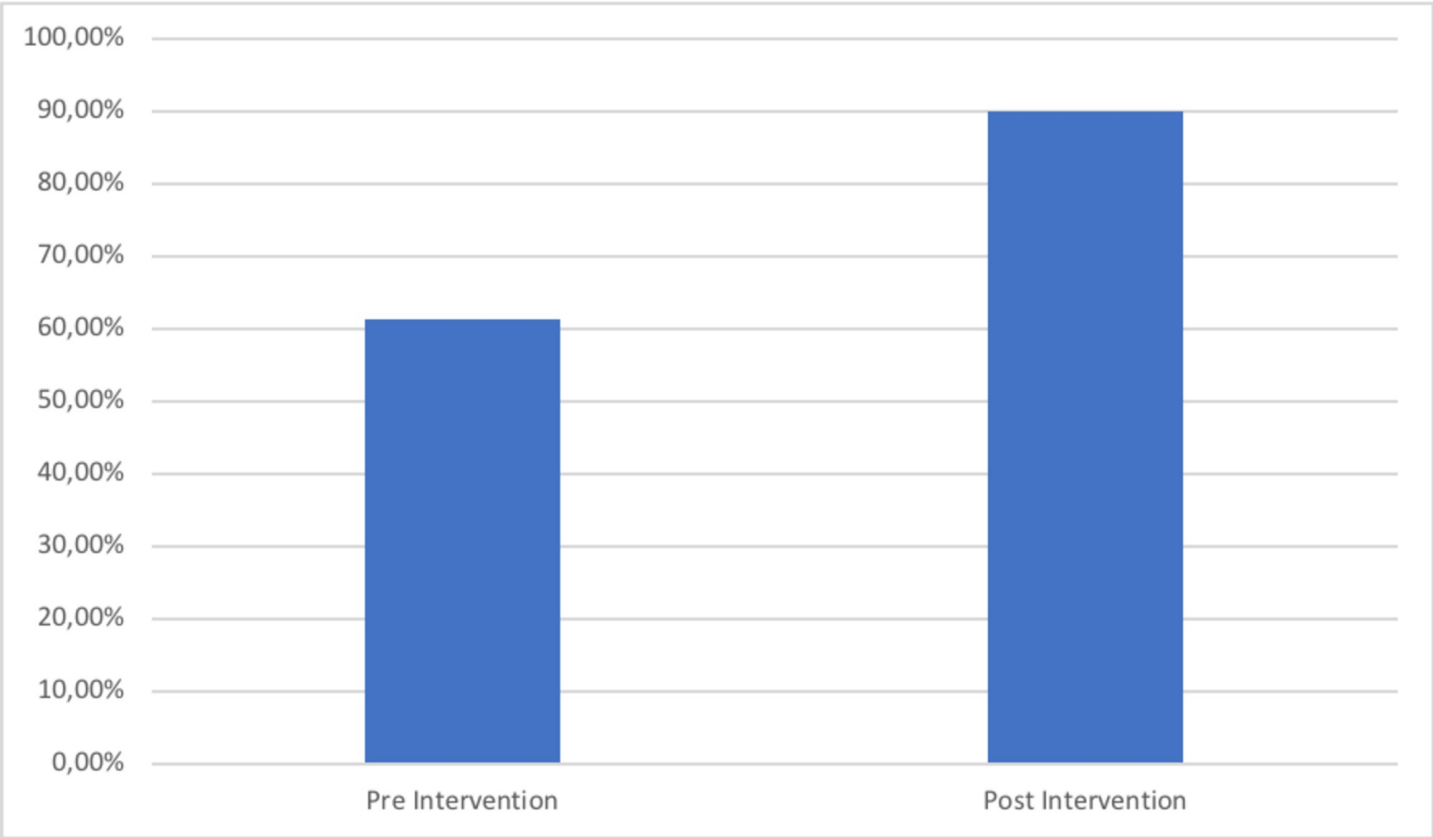
Percentage of patients with day one post-operation osteoporosis bloods requested

The second cohort had a total of 30 patients. Our re-audit demonstrated an improvement of 23% in those achieving all nine criteria of the BPT, from 38.7% (12/31) to 63.3% (19/30) (Figure 2). The NHFS was recorded in 66.7% (20/30) of cases (Figure 3) and there was an increase in the number of patients who had day one post-operative osteoporosis bloods from 61.3% (1/319) to 90% (27/30) (Figure 4). This improvement in the number of patients achieving the BPT, led to a projected uplift payment of £3,045 over the audit period of two months. Extrapolated over a one-year period, this equated to projected uplift payments of £18,270 as a direct consequence of these interventions. The total costings for the implementation of interventions following cycle one of the audit amounted to £2.00. 

## Discussion

This double-loop quality improvement audit analysed factors associated with deficiencies in neck of femur fracture care, and implemented simple and cost-effective interventions to address these deficiencies. Our initial audit highlighted failures in adherence to both the BPT and ERP, with only 38.7% of patients admitted with neck of femur fractures achieving all criteria for the BPT. None of the patients admitted had a NHFS calculated, and 61.2% of patients had day one post-operative bone health bloods checked. As a result of our simple interventions there was a significant improvement in adherence to both the BPT and the ERP, with projected tariff payments of £2,871 over the audit period, which could lead to potential completed tariff payments of £18,270 per annum. 

The National Hip Fracture Database publishes yearly figures, both as national averages and for individual trauma centres, showing trends for key indicators including 30-day mortality, time to surgery, input from geriatricians and the level of BPT attainment [2]. In the year in which our audit was undertaken, the UK national average percentage of patients achieving the BPT was 59.2% [9]. The most recent NHFD report was published in 2019 and represents the statistics for 2018. It demonstrated a national average BPT attainment rate of 58% [2]. 

The BPT was established in order to reduce the high mortality and morbidity rates associated with neck of femur fractures. An increased BPT attainment is thought to directly reduce mortality and morbidity rates in these patients [2]. A 2017 observational cohort study by Oakley et al. based on 2541 patients aged over 60 admitted to a trauma centre with neck of femur fractures, found that there was a reduction in 30-day mortality from 21% in those who did not meet the BPT criteria to 6% in those who did. This was also reflected in longer-term survival figures, with a one-year mortality rate of 28.6% in those who met the tariff criteria and 42% in those who did not [10]. Since March 2012, the published NHFD 30-day mortality rates have reduced from 8.2% to 6% in April 2020 [11]. In recognition of the positive impact this has had for the frail, elderly trauma patient, as of 1st April 2020, patients with femoral shaft and distal femoral fractures will now be incorporated into the BPT, in an effort to attempt to improve patient outcomes for this subgroup [12]. At date of publication, patients with periprosthetic fractures remain outside of the BPT remit.

Since its inception in the early 2000s, the ERP has been introduced to a variety of different surgical specialties in the UK. It has been particularly popular in patients undergoing surgery for colorectal cancer, and in hip and knee arthroplasty in orthopaedics [13,14]. Although minor adaptations may be made to the ERP depending on the requirements of the pathologies it is set up to encompass, the emphasis remains on the optimization of the patient from the outset of admission. In elective procedures this starts prior to admission. In neck of femur fracture patients, the ERP has been shown to reduce the length of stay from 8.21 days to 5.82 days as reported in Kang et al.’s 2019 non-concurrent cohort study which compared the outcomes of 100 patients with intertrochanteric hip fractures; 50 admitted under an ERP and 50 in a control group [7]. This reduction in length of stay is also demonstrated in Liu et al.’s 2017 non-concurrent cohort study of 5002 patients with hip fractures who were admitted to 20 treating centres in the United States. Of these, 2488 were admitted in the 12 months prior to the introduction of an ERP protocol and 2514 in the 12 months following. The length of stay reduced to 3.2 days post-ERP introduction from 3.6 days pre-ERP. Liu et al. also demonstrated a statistically significant reduction in time to first mobilisation, with 21.2% of patients who had been admitted under the ERP able to mobilise within 12 hours of surgery, in comparison to 2.8% of those who had been admitted prior to its introduction [15]. In a similar manner to the ERP at our trust, the components of the ERP detailed in these studies focus on the prescription of appropriate analgesia and pre- and post-operative nutrition.

Since its introduction the BPT has had several revisions, as detailed above. This is unsurprising as clinical pathways are prospectively assessed and adapted to the needs of the patient group. Methods of improvement which can be introduced quickly and without incurring too much expense are invaluable to both clinicians and patients. Our study utilises simple, cost-effective measures which can be easily adapted to aid clinicians to continue to meet the criteria for both the BPT and the ERP as each pathway continues to evolve, and thus ensure a high standard of patient care. The strength of our study is that it demonstrates how these simple measures can have a significant impact on the quality of care that patients receive. Neck of femur fracture patients are often complex, multi-morbid patients, with a higher number of clinical aspects to their care, presenting more of a challenge for the clinical team. As advised by NICE, it is vital that they are managed by a multi-disciplinary team, with clearly outlined criteria to achieve in order to obtain the optimum patient outcome [11]. 

The processes of clinical audit and quality improvement are integral to modern healthcare. Clinical audit was first integrated into clinical governance systems in 1997 [16] and involves the evaluation of local practice against existing standards aiming to ensure high-quality patient care is delivered. Regular clinical audit is now a national requirement in the United Kingdom [16]. Both the Enhanced Recovery Programme and the Best Practice Tariff are national standards, which undergo regular audit on both a national and local level. The aforementioned National Hip Fracture database is an annual national audit of the management of patients in the UK with hip fractures and any changes made to the BPT are incorporated into the remit of the database.

Our study has limitations. The patient populations are small, with a maximum of 31 patients per cohort. Whilst the savings for each audit cycle period are recorded, the estimated yearly savings are extrapolations, and rely on the admitting teams continuing to meet the ERP and BPT at the level demonstrated during the second audit cycle. Due to the rotation-based nature of junior doctor training, following audit completion it is often difficult to continue to maintain standards achieved when the teams involved have moved on and subsequent cohorts require further training. However, we believe that we have illustrated how simple interventions can significantly improve patient care, reduce mortality, and lead to increased savings for hospitals. 

## Conclusions

Interventions that simplify requesting processes and place ownership of a task at a time-specific juncture in a patients’ journey will significantly improve the ability of the pathway to succeed. Our audit has shown that educational training and simple cost-effective measures can be employed to improve care for these complex patients. In addition to this, it demonstrates how technology can be utilised in clinical practice, to significantly increase clinical efficiency.
